# The histopathological spectrum of myocardial inflammation in relation to circumstance of death: a retrospective cohort study in clinical and forensic autopsies

**DOI:** 10.1080/20961790.2021.1989793

**Published:** 2021-11-25

**Authors:** Romy du Long, Judith Fronczek, Hans W. M. Niessen, Allard C. van der Wal, Hans H. de Boer

**Affiliations:** a Department of Pathology, Amsterdam University Medical Center, University of Amsterdam, Amsterdam, The Netherlands; b Department of Forensic Medicine, Netherlands Forensic Institute, The Hague, The Netherlands

## Abstract

Interpreting a myocardial inflammation as causal, contributory or as of no significance at all in the cause of death can be challenging, especially in cases where other pathologic and/or medico-legal findings are also present. To further evaluate the significance of myocardial inflammation as a cause of death we performed a retrospective cohort study of forensic and clinical autopsy cases. We revised the spectrum of histological inflammatory parameters in the myocardium of 79 adult autopsy cases and related these to the reported cause of death. Myocardial slides were reviewed for the distribution and intensity of inflammatory cell infiltrations, the predominant inflammatory cell type, and the presence of inflammation-associated myocyte injury, fibrosis, edema and hemorrhage. Next, the cases were divided over three groups, based on the reported cause of death. Group 1 (*n* = 27) consisted of all individuals with an obvious unnatural cause of death. Group 2 (*n* = 29) included all individuals in which myocarditis was interpreted to be one out of more possible causes of death. Group 3 (*n* = 23) consisted of all individuals in which myocarditis was reported to be the only significant finding at autopsy, and no other cause of death was found. Systematic application of our histological parameters showed that only a diffuse increase of inflammatory cells could discriminate between an incidental presence of inflammation (Group 1) or a potentially significant one (Groups 2 and 3). No other histological parameter showed significant differences between the groups. Our results suggest that generally used histological parameters are often insufficient to differentiate an incidental myocarditis from a (potentially) significant one.

## Keypoints

- Determining the significance of myocardial inflammation for the cause of death can be challenging.
- Our study reviewed the histological spectrum of myocardial inflammation between three groups of autopsy cases, defined by their reported cause of death.
- Only a diffuse increase of inflammatory cells could reliably discriminate between an incidental presence of inflammation or a potentially significant one.
- Determining the cause of death in case of myocardial inflammation requires a comprehensive approach.

## Introduction

Myocarditis or myocardial inflammation is a common finding in forensic and clinical autopsies, with incidences of myocarditis in autopsy studies of sudden cardiac death ranging from 0.3% to 14.8%, including infants, children and adults [1–5]. It is however long recognized that the histopathological diagnosis of myocarditis is challenging [5–8]. Besides that, the clinical presentation of myocarditis is highly variable, ranging from subclinical “flu-like” symptoms to sudden death [6,9,10]. This may complicate the determination of the cause of death in case of myocardial inflammation and myocarditis considerably.

Myocarditis may be listed as a possible, a probable or even the most likely cause of death, depending on the extent of the inflammation, and the presence of myocyte injury. This approach is in line with the current guidelines for the histopathological diagnosis of myocarditis in a clinical context, and endorsed by the Association of European Cardiovascular Pathology (AECVP) [11]. According to their most recent guideline, “fulminant” or “multifocal” myocarditis can be generally considered as a reliable or acceptable cause of death, especially when it is the only substantial (histo)pathological finding. More challenging however are cases with less severe or even sparse myocardial inflammation; its significance is then far less certain. In those cases, pathologists are advised to carefully screen for, and exclude other (cardiac and non-cardiac) causes of death before the limited inflammation can be suggested as the cause of death. Depending on the context of the case, such screening could focus on genetic screening for familial arrhythmia syndromes, toxicological analysis and signs of a violent cause of death.

Due to its comprehensiveness, such a screening may aid in the eventual determination of the cause and manner of death. However, especially cases with other non-conclusive but medico-legally significant findings or circumstances can lead to difficulties in interpreting the role of myocarditis as causal, contributory or as of no significance at all in the cause of death. For instance, myocarditis may be found in cases in which the autopsy results are also befitting a diagnosis of strangulation, or when the circumstances of death are suggestive of restraint asphyxia. In addition, myocardial inflammation may co-occur with toxicological results of borderline lethality.

A more detailed study on correlations between the patterns of myocardial inflammation and distinct categories of causes of death could provide additional information on the significance of myocarditis as a cause of death. In this study we therefore investigated the spectrum of histological (inflammatory) infiltrations in the myocardium in an adult (forensic and clinical) autopsy population, and subsequently related these findings to the reported cause of death, retrieved from the autopsy reports. Specifically, the cases were grouped into three groups: (1) sudden and obvious non-natural deaths; (2) myocarditis as one of at least two potential causes of death; (3) myocarditis reported as the only significant finding. By doing so, this study aims to advance our knowledge on the significance of myocardial inflammation and myocarditis in determining the cause of death.

## Materials and method

### Study design and population

This study is a retrospective, double-centre study including both forensic and clinical autopsy cases. All cases of the Netherlands Forensic Institute (NFI) and the Amsterdam University Medical Center (Amsterdam UMC) from January 1st 2014 until December 31th 2019 were reviewed and used for this study with institutional approval.

The cases from the Amsterdam UMC were retrieved by a search in the Dutch National Database of Pathology (Pathologisch-Anatomisch Landelijk Geautomatiseerd Archief, PALGA), which mandatorily lists all diagnoses in a standardized way. All cases with “myocarditis” or “myocardial inflammation” in their diagnosis were included. These cases also included cases for which an expert opinion was requested from other Dutch hospitals. For the NFI, all autopsy reports were reviewed and all cases with “myocarditis” or a mention of myocardial inflammation in the diagnosis, conclusion text or histological report were included. The search yielded a total of 85 forensic cases and 51 clinical cases. Note that these cases are not limited to myocarditis, but that all cases with some form of myocardial inflammation were included.

All underage individuals (age <18 years) or individuals with a known systemic disease (e.g. rheumatoid arthritis, sarcoidosis, systemic lupus erythematosus) or ischemic cardiac disease (e.g. acute, healing or healed myocardial infarction) were excluded. Cases with incomplete autopsy reports or paraffin blocks of the heart were also excluded.

Eventually, a total of 79 cases with myocardial inflammation remained in the study. These consisted of 41 forensic cases and 38 clinical cases, with a total of 56 males and 23 females.

### Data collection

For all 79 cases, the number and locations of the myocardial formalin-fixed paraffin-embedded hematoxylin and eosin (HE) slides were recorded. All myocardial slides per case were reviewed for various histological features:

First, the distribution of the inflammatory infiltrations was studied. For this purpose, the number and location of inflammatory foci was analyzed in the HE slides. A focus of inflammation was defined as ≥10 inflammatory cells, as applied also previously in other autopsy studies [5,12]. Cases without distinct foci, but with a diffuse presence of inflammatory cells were listed as “diffuse”.

**Figure 1.**
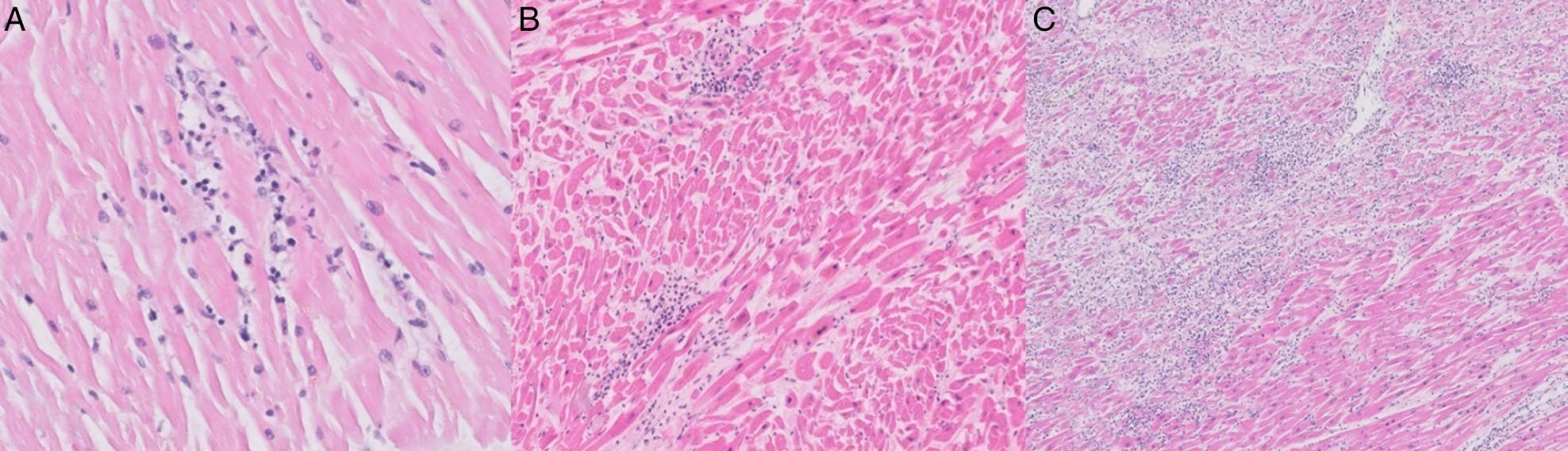
Micrographs with examples of a various types of myocardial inflammation. (A) A single focus of inflammatory cells with myocyte injury, constituting a focal myocarditis (HE, ×20). (B) Two inflammatory foci with myocyte necrosis, consistent with a diagnosis of multifocal myocarditis (HE, ×10). (C) Extensive diffuse myocardial inflammation with myocyte injury, i.e. an active diffuse myocarditis (HE, ×5).

Second, the severity of myocardial inflammation per case was determined by calculating the inflammatory index (total number of foci divided by the total number of slides) according to Kitulwatte et al. [13]. According to that paper, scores should be interpreted as no (0), scant (0.1–1.0), mild (1.1–1.9), moderate (2.0–4.9) and marked (≥5.0) inflammation. The severity of the inflammation was furthermore classified based on the subdivision in Basso et al. [11,14], using the following definitions (Figure 1):

- Borderline myocarditis: sparse/“starry sky” foci of ≥10 inflammatory cells *without myocyte injury* in any of the sections;
- Focal myocarditis: at least one focus of ≥10 inflammatory cells *with myocyte injury* in any of the sections;
- Multifocal myocarditis: more than one focus of ≥10 inflammatory cells in one section or at least one focus of ≥10 inflammatory cells *with myocyte injury* in multiple sections;
- Acute diffuse myocarditis: diffuse inflammatory infiltrations of inflammatory cells *with widespread myocyte injury* in any of the sections.

**Figure 2.**
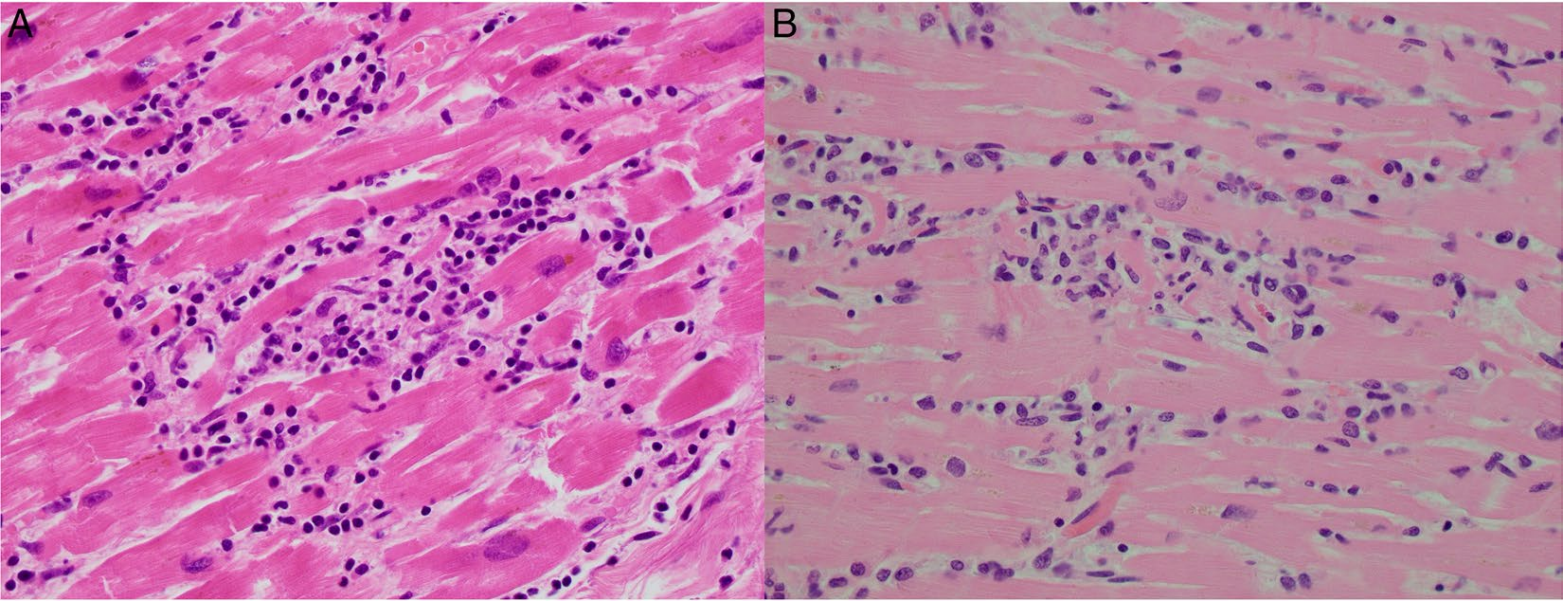
Micrographs with examples of myocyte injury. (A) An almost totally disintegrated cardiomyocyte amidst an inflammatory infiltrate consisting of lymphocytes and macrophages (HE, ×20). (B) The necrotic cardiomyocyte is still recognizable as thin, slightly eosinophilic cell remnants, adjacent to normal, vital cardiomyocytes (HE, ×20).

Myocyte injury was defined as vacuolization, fragmentation or disintegration of one or more cardiomyocytes in the presence of inflammatory cells. (Figure 2).

**Figure 3.**
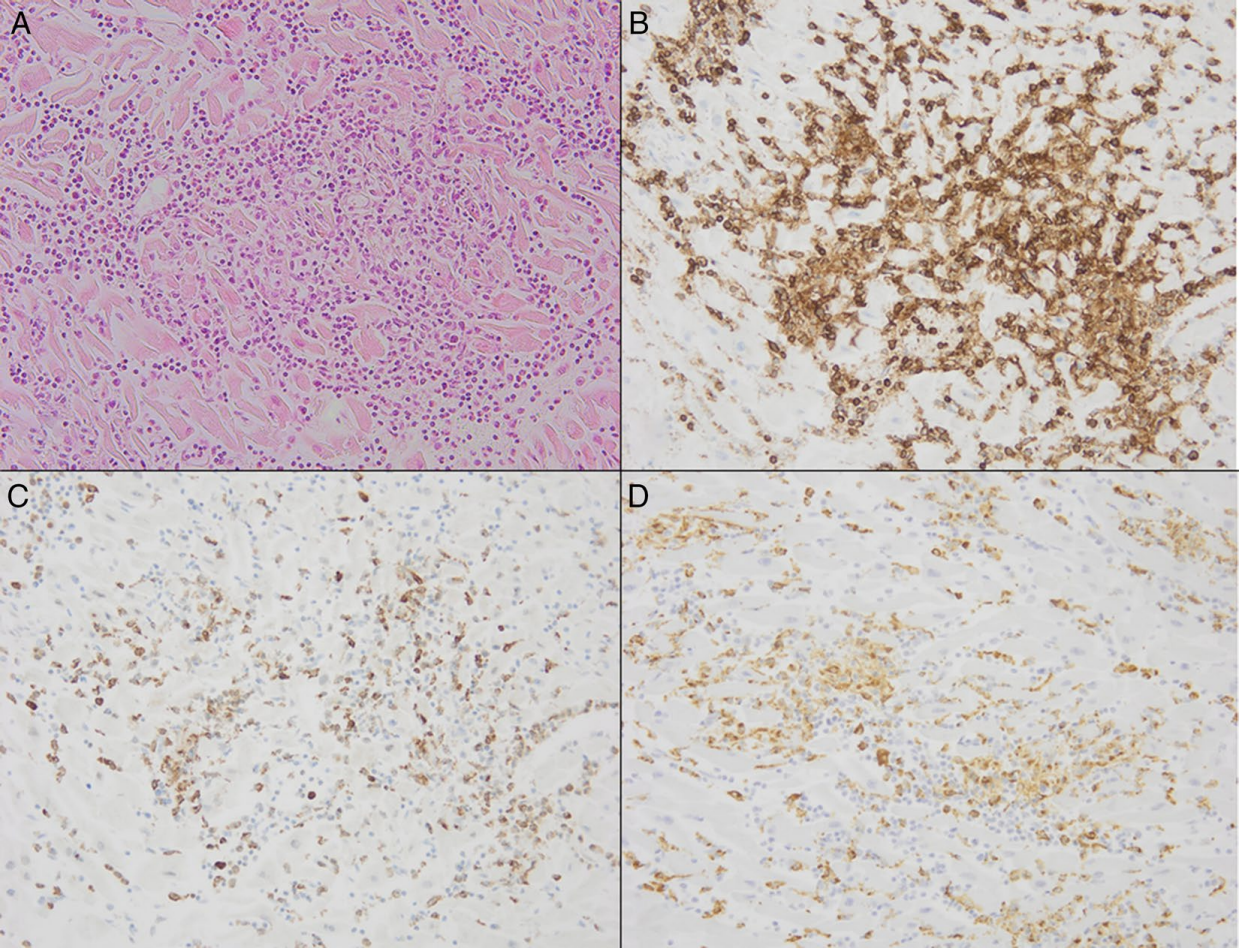
Micrographs of a case of fulminant eosinophilic myocarditis, to illustrate the application of immunohistochemical staining. (A) The standard haematoxylin and eosin-stained section (HE, ×10) shows abundant inflammation, (B) which consists of CD45-positive lymphocytes (immunohistochemical staining, ×10), (C) CD68-positive macrophages (immunohistochemical staining, ×10) and (D) myeloperoxidase-positive neutrophilic and eosinophilic granulocytes (immunohistochemical staining, ×10).

Third, the predominant infiltrative cell type per case was determined by revision of the HE and immunohistochemical stains CD45 (lymphocytes), CD68 (macrophages) and myeloperoxidase (MPO; neutrophils) (Figure 3). All cases included these immunohistochemical stains.

Lastly, the presence of myocyte injury, fibrosis, edema and hemorrhage per case was reviewed. These were scored binary (either absent or present), based on the severest degree per case.

### Intra-observer agreement

All histological re-examination was performed by one researcher (RdL). To test the intra-observer agreement for the number of foci; the predominant infiltrate cell type; and the presence of myocyte injury, fibrosis, edema and hemorrhage; the histological slides were re-examined a second time at least 4 weeks after the first examination.

### Classification of cases

For the comparison of the results of the histological re-evaluation, all cases were classified based on their presumed cause of death, which was adopted unchanged/without revision from the autopsy reports. This reported cause of death was based on the available forensic and/or clinical data, the autopsy results and ancillary postmortem investigations (i.e. toxicology). No additional testing was performed for the purpose of this study. See Table 1 for details per group. All autopsies included a full external and internal examination, including histology of all major organs.

**Table 1.** Clinical characteristics of the study population (*N *= 79).

Parameter	**Group 1** (*n* = 27)	**Group 2** (*n* = 29)	**Group 3** (*n* = 23)
*Age (year)*			
Mean±SD	38.4±14.7	56.7±18.3	40.4±14.8
Range	18–76	22–91	20–69
*Sex*			
Male	24	19	13
Female	3	10	10
*Context of autopsy*			
Forensic	27	8	6
Clinical	–	21	17
**Causes of death**			
***Forensic cases***			
Stabbing	8	–	–
Ballistic trauma	8	–	–
Blunt force trauma to the head	3	1	–
Strangulation	–	1	–
High velocity impact injury	3	–	–
Toxicology	2	3	–
Thermal injury	3	–	–
***Clinical cases***	–	3	–
Pneumonia			
Pulmonary embolism	–	5	–
Sepsis	–	5	–
Myocarditis	–	29^a^	23
Non-ischemic cardiac disease	–	4	–
(other than myocarditis)^b^			
Other^c^	–	7	–

a In all cases in Group 2 the possible cause of death was a combination of myocardial inflammation and the mentioned cause of death.

b Non-ischemic cardiac disease includes hypertrophic cardiomyopathy, HCM (*n*=1), dilated cardiomyopathy, DCM (*n*=2) and arrhythmogenic cardiomyopathy, ACM (*n* =1).

c Subarachnoid bleeding (*n* = 2), ischemic colitis (*n* = 1), chronic obstructive pulmonary disease, COPD (*n*=2), iatrogenic/surgical (*n* = 1) and metastatic esophageal carcinoma (*n* = 1).

Group 1 (*n* = 27) consisted of all patients who died of an obvious unnatural cause of death, with a survival time of less than 1 h. This group included for instance lethal stabbings, shootings or high velocity traumas (e.g. airplane crashes or car accidents). In these cases, the myocardial inflammation was deemed an incidental finding, inconsequential for the cause of death.

Group 2 (*n* = 29) consisted of all patients in whom myocardial inflammation was deemed one out of more possible causes of death. This group for instance included cases where the autopsy results did not allow for a definitive differentiation between a death due to myocarditis or toxicology.

Group 3 (*n* = 23) consisted of all patients in whom myocardial inflammation was the only significant histological finding during autopsy whereas no other (histo)pathological cause of death was mentioned in the autopsy report. None of the individuals in Groups 2 and 3 underwent successful resuscitation.

### Statistical analysis

The intra-observer agreement was calculated with Cohen’s kappa (κ), with a κ > 0.80 considered to represent a strong level of agreement. The results of the histological re-evaluation per group were compared using a Chi-square test, and a Fisher’s exact test when any of the three groups had less than five cases. If there was no normal distribution of scores based on the Chi-square test between the groups, a Kruskal-Wallis H test was used to determine statistical significance. A *P*-value of <0.05 was considered significant. All analyses were performed using SPSS version 26 (https://www.ibm.com/support/pages/downloading-ibm-spss-statistics-26).

## Results

### Intra-observer agreement

There was a good intra-observer agreement for the number of foci on the HE slides (*κ* = 0.85) and for scoring myocyte damage (*κ* = 0.96), edema (*κ* = 1.00), fibrosis (*κ* = 0.88) and hemorrhage (*κ* = 0.92).

### Myocardial sampling

The number and location of the histological slides sampled per group is shown in Table 2. Group 1 showed the lowest mean number of sampled locations (5.6); for Groups 2 and 3 this was 8.3 and 7.8 respectively. These differences were only significant between Groups 1 and 2 (χ^2^ (3) = 28.010, *P* = 0.000). Although Group 2 and Group 3 presented the highest mean number of slides, these groups also showed the largest variation in the number of sampled locations. Almost all cases included at least samples of the right ventricle, left ventricle (anterior, lateral and posterior wall) and ventricle septum. More extensive sampling generally included slides from the left and right ventricle, sometimes in combination with the atria. The conduction system was sampled in 13 cases.

**Table 2.** Sampling of cardiac locations *(N = 79)*.

Parameter	**Group 1** (*n* = 27)	**Group 2** (*n* = 29)	**Group 3** (*n* = 23)
**Number of cardiac locations sampled**			
Mean±SD	5.6±1.3	8.3±3.7	7.8±4.0
Range	5–10	3–18	1–16
**Number of slides per location**			
Right ventricle	27	27	21
Left ventricle			
Anterior wall	27	29	23
Lateral wall	27	29	22
Posterior wall	27	29	22
Ventricle septum	27	27	21
Right atrium	–	9	6
Left atrium	–	9	7
Conduction system (total)	1	7	5
SA-node	1	7	–
AV-node	1	3	5

### Distribution of inflammation

The type and location of the inflammation per group are shown in Table 3. In 17 out of 79 cases (21.5%) the cardiac inflammation was diffuse. This type of inflammation was mostly found in Groups 2 and 3 (9 and 7 cases respectively), and only once in Group 1.

**Table 3.** Histological assessment of myocardial inflammation per group (*N* = 79).

Parameter	**Group 1** **(*n* = 27)**	**Group 2** **(*n* = 29)**	**Group 3** **(*n* = 23)**	*P*-value
**Number of cases with diffuse inflammation**	1	9	7	0.021
**Number of foci^a^**				
Mean	2.3	2.6	1.8	0.375
Range	1–12	1–11	1–7	
**Cardiac locations with inflammation**				
** **Right ventricle	5/27	10/27	7/21	1.000
Left ventricle				
Anterior wall	10/27	13/29	9/23	0.672
Lateral wall	8/27	13/29	10/22	0.886
Posterior wall	7/27	11/20	7/22	0.939
Ventricle septum	9/27	10/27	8/21	0.433
Right atrium	–	0/9	1/6	1.000
Left atrium	–	0/9	1/7	0.154
Conduction system	0/1	2/7	1/5	0.375
**Inflammatory index^b^**				
No (0)*	1	9	7	0.021
Scant (0.1–1.0)	24	17	16	0.067
Mild (1.1–1.9)	2	3	–	0.419
Moderate (2.0–4.9)	–	–	–	–
Marked (≥5.0)	–	–	–	–
**Severity of inflammation^c^**				
Borderline myocarditis	6	7	7	0.564
Focal myocarditis	10	4	5	0.121
Multifocal myocarditis	10	9	4	0.150
Diffuse acute myocarditis	1	9	7	0.021
**Infiltrate type**				
Lymphocytic	26	13	18	0.418
Neutrophilic	–	2	–	0.220
Eosinophilic	–	3	2	0.748
Mixed	1	10	3	0.080
Giant cell	–	1	–	0.399

a Cases with diffuse inflammation excluded.

b According to Kitulwatte et al. [13].

c Adapted from Basso et al. [11, 14] with permission.*Cases with diffuse acute myocarditis.

All other cases showed inflammatory foci with, interestingly, little difference between the various groups in terms of number and distribution. The mean number of foci per case was highest in Group 2 (2.6) and lowest in Group 3 (1.8), but this difference was not statistically significant (*P* = 0.375), and all groups showed a wide distribution. Foci were most often found in the left ventricle wall, with a more or less even distribution over the various locations. Cases in Group 2 showed the largest distribution of foci in the myocardium, showing almost equal involvement of the right ventricle, left ventricle and ventricle septum. In three of the 13 cases where the conduction system was sampled, foci were also found there. The atria were both affected in just one case.

### Severity of the inflammation

Applying the inflammatory index of Kitulwatte et al. [13] to our dataset yielded the following results. Due to lack of foci, the inflammatory index in cases with diffuse inflammation was 0. In all groups, most cases showed “scant” inflammation (24/27 in Group 1, 17/29 in Group 2 and 16/23 in Group 3). “Mild” inflammation was least noted. “Moderate” and “marked” inflammation were not seen in any of the cases (Table 3).

Also shown in Table 3 are the results when applying the classification of Basso et al. [11,14]. The number of “borderline-“, “focal-“ or “multifocal myocarditis” were not significantly different (*P* = 0.564, 0.121 and 0.150). The only obvious and statistically significant difference was found in the number of diffuse acute myocarditis, which was almost invariably found in Groups 2 and 3 (9 and 7 cases, *vs.* 1 in Group 1; *P* = 0.021).

### Type of inflammatory infiltrate

Almost all infiltrates in Group 1 were predominantly lymphocytic (26/27), and one case showed a mixed inflammatory infiltrate. Also in Group 3, the large majority (18/23) was predominantly lymphocytic; three cases showed mixed inflammation and two cases were deemed eosinophilic. The inflammatory infiltrates were most varied in Group 2, with only about half (13/29) being lymphocytic. This group also included mixed, neutrophilic, eosinophilic or giant cell type inflammation (Table 3).

### Histological features

As shown in Table 4, there were no statistically significant differences in the presence of myocyte injury, edema, fibrosis or hemorrhage. Myocyte injury was more or less equally distributed over the three groups (78%, 76% and 70%, respectively). Edema was seen in 16 out of 29 cases (55%) in Group 2 compared to 9 out of 27 (33%) and 9 out of 23 cases (39%) in Groups 1 and 3. Fibrosis and hemorrhage was only seen in a small proportion of cases.

**Table 4.** Histological features (*n*, %) within the three groups, based on Haematoxylin and Eosin slides (*N*=79).

Histological features	**Group 1** **(*n* = 27)**	**Group 2** **(*n* = 29)**	**Group 3** **(*n* = 23)**	*P*-value
**Myocyte injury**	21 (78)	22 (76)	16 (70)	0.950
**Edema**	9 (33)	16 (55)	9 (39)	0.137
**Fibrosis**	1 (4)	4 (14)	0 (0)	0.101
**Hemorrhage**	1 (4)	1 (3)	1 (4)	0.534

## Discussion

Due to the clinical and histopathological heterogeneity of myocarditis, pathologists have long been searching for features that help to differentiate an incidental myocardial inflammation from one with (potential) significance to the cause of death. Although histological examination is a *sine qua non* for the diagnosis of myocarditis, our results suggest that the studied histological features alone do not necessarily allow for a determination between an incidental and a potentially significant one.

Only the presence of a diffuse type of inflammation differed significantly between Group 1 (sudden non-natural death) and Group 2 or 3 (myocarditis potentially related to the cause of death), and thus could more or less reliably differentiate between an incidental or a potentially significant myocardial inflammation in the deceased. Non-diffuse, (multi)focal inflammation was found frequently in all three groups, with no obvious difference in the mean number or range of observed foci. Our results therefore show that even a considerable number of inflammatory foci can be an incidental finding, as represented by cases in Group 1. At the same time, in a few cases, a single inflammatory focus was the only histopathological finding in an otherwise unexplained death (Group 3). However, this latter finding must be interpreted with caution (see below).

The severity of the inflammation is considered by many investigators to be helpful to discriminate between inconsequential or significant inflammation, since a widespread inflammation is assumed to be related to more severe clinical symptoms or an increased risk of arrhythmias [11,15,16]. This rationale is also reflected in the proposed inflammatory index by Kitulwatte et al. [13]. In their study, the majority of cases with assumed fatal myocarditis in their study showed marked or moderate inflammation. However, less severe inflammation was also found. Kitulwatte et al. [13] stated that these individuals may represented a “potentially over-diagnosed population where another non-obvious cause of death should also be considered” and concluded that “microscopically significant number of foci of myocyte necrosis and surrounding inflammation are the essential features of myocarditis”.

When we applied the inflammatory index in our study, it was unable to reliably differentiate between our three groups, with the majority of cases showing low inflammatory indices. In addition, our results indicate that even in cases with an obvious alternative cause of death, scant or even mild inflammation can be found. As a result, the approach of Kitulwatte et al. [13] seems to be most informative in case of higher inflammatory indices. Lower inflammatory indices seem to be of limited value. It must be kept in mind that the inflammatory index should be applied only to non-diffuse types of inflammation. A diffuse type of inflammation, being the severest form, shows by definition no distinct inflammatory foci, and therefore scores an inflammatory index of 0. This is misleading as it inappropriately suggests a lack of inflammation.

The potential relevance of the intensity of the inflammation was furthermore studied following the guidelines of the AECVP reported by Basso et al. [11,14]. In our study the severest form, “acute diffuse myocarditis” was almost invariably found in Groups 2 and 3. Given the single case of “acute diffuse myocarditis” in Group 1, one could argue that such a finding still does not provide certainty on the cause of death. Autopsy bias must however be considered: it cannot be excluded that this one person would have died due to myocarditis if the fatal, non-natural event would not have occurred. Therefore, in line with the guidelines of the AECVP, we agree that acute diffuse inflammation with extensive myocyte injury, in a proper clinical context, could be interpreted as a “certain” cause of death. Multifocal, focal and borderline myocarditis were found more or less evenly distributed over the three groups, indicating that these types can be both incidental and potentially relevant.

The results pertaining to the type of inflammatory infiltrate show that incidental myocardial inflammation is almost invariably lymphocytic. The value of this observation is however limited, as this type of inflammation was also the most frequent in groups 2 and 3. Based on our results, a non-lymphocytic predominant infiltrate seems to support a potentially relevant cardiac inflammation. However, the limited number of such cases, and the limitations of our study (see below) preclude further statements hereon.

Myocyte injury, edema, fibrosis and hemorrhage were also evenly distributed over the three defined groups. As such, none of these histological features seems useful in isolation to unequivocally differentiate between an incidental and potentially relevant cardiac inflammation. Myocyte injury is historically considered a feature that differentiates between a significant and non-significant myocardial inflammation [17]. For instance, the Dallas criteria [17] and the guidelines of the AECVP consider it a necessary feature of myocarditis, but not specifically related to onset of death. In practice, the Dallas criteria are most frequently applied on endomyocardial biopsies of living patients. In a more recent study by Casali et al. [10], myocyte injury was deemed a significant finding to distinguish an incidental from a lethal myocarditis. This contrasts with our findings, which suggests that myocyte injury can be an incidental finding, not directly related to the cause of death.

Our study design allows for relatively robust statements on the histological spectrum of “incidental” myocardial inflammation. However, several limitations make conclusions pertaining to Groups 2 and 3 more difficult. None of these cases included genetic testing or family histories to evaluate a possible underlying genetic predisposition to a ventricular arrhythmia (e.g. channelopathies). A toxicological cause of death was ruled out for all forensic cases in Groups 2 and 3, but not for the clinical autopsies. Furthermore, the co-morbidities in Group 2 may also have affected the presence and/or severity of the occurrence of myocarditis, e.g. in case of sepsis or pneumonia. This further impeded the interpretation of the myocardial inflammation for the mechanism and cause of death. A prospective study design could have helped to mitigate these limitations. All in all, the significance of the myocardial inflammation may have been overestimated in some of the cases in Groups 2 and 3.

This potentially overestimation of myocardial inflammation as the cause of death, especially in the absence of other histopathological findings, and auxiliary toxicology/genetic testing, was also identified earlier by Bonsignore et al. [8]. Such an incomplete diagnostic workup may have important negative implications. In a forensic context, overinterpretation of myocardial inflammation may unnecessarily complicate the judicial process, or in the most extreme sense, exonerate a suspect inappropriately. In a clinical setting, a diagnosis of myocarditis usually concludes the investigation. Especially in cases of sudden, unexpected and otherwise unexplained death, a genetic or toxicological cause may remain unnoticed because of this. In case of the former, such a discovery may eventually be lifesaving for relatives of the decedent. This was emphasized previously by Papadakis et al. [18], who showed that sudden cardiac death victims with “minor” cardiac abnormalities—such as a focal or borderline myocarditis—were as likely to harbour an inheritable cardiac disease as those sudden cardiac death victims with totally normal hearts at autopsy (51% *vs*. 47%). Our study, despite its limitations, does add significantly to this discussion, by showing that myocardial inflammation—even with myocyte necrosis—may be an incidental finding. This should prompt a low threshold for auxiliary genetic and toxicological testing. The study of Tan et al. [19] emphasizes the high prevalence of genetic abnormalities when sudden death remains unexplained. In their study, 40% of the individuals who died suddenly and unexpectedly (age <40 years), had an inheritable cardiac disease that explained death. Most of the hearts in that study were structurally normal.

All in all, our results indicate that histology alone may be insufficient to differentiate an incidental myocarditis from a (potentially) significant one. Perhaps more detailed methods, as developed for myocardial biopsies from living patients [20,21] may be able to increase the specificity and sensitivity of the histological analysis and provide additional insights in its relevance to the cause of death. Virology testing combined with a detailed examination of the conduction system may also be of help, to examine the possibility of a fatal cardiac arrythmia in the context of myocardial inflammation. Still, the probability of death due to myocarditis will often also depend on the extent in which other causes of death have been excluded. This prompts an extensive investigation into the cause of death. Per the guidelines of the AECVP, this should not be limited to a full external and internal examination with histology of the major organs. It should also include a review of the clinical history, a thorough study of the circumstances surrounding death, biochemistry, toxicology, and genetic counselling to identify (familial) arrhythmic disease. Microbiology and virology should also be considered. When circumstances render such a comprehensive investigation impossible, it appears best to be cautious with definitive statements pertaining to the cause and mechanism of death.

## Conclusion

The results of our retrospective analysis of 79 clinical and forensic autopsies indicate that the histological spectrum of myocardial inflammation varies considerably. Besides a diffuse, acute myocardial inflammation with wide-spread myocyte injury, none of the studied histological features could reliably distinguish between our clinical groups, and thereby between an alleged incidental and potentially lethal myocardial inflammation. Determining the significance of myocardial inflammation for the cause of death usually requires an extensive analysis, based on all autopsy findings, the results of additional tests (e.g. toxicology, virology, genetic testing), and a thoroughly study of the context of the case.

## Authors’ contribution
